# Effects of Exercises of Different Intensities on Bone Microstructure and Cardiovascular Risk Factors in Ovariectomized Mice

**DOI:** 10.3390/ijms26031005

**Published:** 2025-01-24

**Authors:** Xiaoni Wang, Yiting Kang, Jie Yao, Xiaohang Gao, Zeguo Feng, Yifei Song, Xiaohui Di, Qianyu Zhang, Jianbao Zhang

**Affiliations:** 1 Key Laboratory of Biomedical Information Engineering of Ministry of Education, Institute of Health and Rehabilitation Science, School of Life Science and Technology, Xi’an Jiaotong University, Xi’an 710049, China; xiaoni.wang@mail.xjtu.edu.cn (X.W.); kyt9449@stu.xjtu.edu.cn (Y.K.); 2041005@sntcm.edu.cn (J.Y.); gaoxiaohang@lcu.edu.cn (X.G.); zeguofeng@stu.xjtu.edu.cn (Z.F.); syf0522@stu.xjtu.edu.cn (Y.S.); dixiaohui@stu.xjtu.edu.cn (X.D.); zhangqianyu@stu.xjtu.edu.cn (Q.Z.); 2 School of Nursing, Shaanxi University of Chinese Medicine, Xianyang 712046, China

**Keywords:** ovariectomized, vascular, bone, moderate-intensity continuous training, high-intensity interval training

## Abstract

Postmenopausal women face increased risks of osteoporosis and cardiovascular diseases due to estrogen decline. This study investigated the effects of moderate-intensity continuous training (MICT) and high-intensity interval training (HIIT) on bone microstructure and cardiovascular risk factors in ovariectomized (OVX) mice. Results showed that both exercise regimens improved blood lipid profiles and vascular structure, reducing systolic blood pressure (−11.81% and −10.89%) and undercarboxylated osteocalcin (ucOCN) levels (−52.14% and −52.05%). However, moderate-intensity exercise was more effective in enhancing bone mineral density (+82.38% and +45.02%) and microstructure recovery. No significant correlation was found between ucOCN and cardiovascular disease risk factors, such as lipid parameters, systolic blood pressure, and vascular wall thickness. This study suggests that both exercise intensities can mitigate cardiovascular risks in OVX mice, which is independent of OCN. MICT is superior for promoting osteoporosis recovery.

## 1. Introduction

Menopause, defined as the cessation of menstruation due to the permanent loss of ovarian follicular function, can see women spending up to 40% of their lives in a postmenopausal state [1,2]. The incidence of osteoporosis and cardiovascular disease (CVD) significantly increases in postmenopausal women [3,4]. Studies have shown a close link between bone health and CVD [5,6]. Low bone mineral density is associated with endothelial dysfunction, coronary artery disease, peripheral vascular disease, and cardiovascular mortality [7,8]. Complications such as fractures caused by osteoporosis and CVD severely diminish the quality of life of patients and are major causes of death among elderly women [9,10,11].

Appropriate exercise can increase bone density and prevent cardiovascular disease [12]. Due to the significant role of exercise in disease prevention and treatment, a review published in 2016 proposed that “exercise is a kind of medicine” [13]. However, it remains unclear whether there are differences in the preventive and therapeutic effects of different exercise intensities on cardiovascular disease and osteoporosis in menopausal women, as well as the underlying mechanisms of these effects.

Osteocalcin (OCN) is a small protein secreted by osteoblasts that not only affects bone formation but also enters the bloodstream to influence glucose metabolism and the cardiovascular system, serving as an important endocrine factor [14]. Moreover, serum OCN is one of the few factors with functions that cover major menopause-related diseases in women, including osteoporosis, cardiovascular disease, and anxiety [15]. Studies on menopausal women have shown that coronary artery calcium score and atherosclerosis are positively correlated with OCN concentrations [16,17], indicating that serum OCN may play a role in cardiovascular diseases among menopausal women. It is necessary to investigate the exercise-induced changes of OCN in order to clarify the roles of exercise on the cardiovascular system.

The OVX mouse model can effectively mimic the changes in estrogen levels in postmenopausal women and has therefore been widely accepted as a model for menopause to study postmenopausal symptoms [18,19]. Therefore, in this study, we used ovariectomized (OVX) mice as a model and subjected them to moderate-intensity continuous exercise and high-intensity interval exercise to examine their effects on serum OCN levels, as well as cardiovascular risk factors and osteoporosis in OVX mice. Our study indicates that there is no difference between the two exercise modalities in improving cardiovascular disease risk factors in OVX mice, with MICT showing superior effects on bone microstructure compared to HIIT. Meanwhile, ucOCN does not appear to be the direct cause of the improvement in cardiovascular risk factors due to exercise; rather, it may be related to changes in estrogen levels. Conversely, ucOCN could serve as a potential biomarker for assessing the effectiveness of exercise in the prevention and treatment of osteoporosis.

## 2. Results

### 2.1. Body Weight and Uterus Weight

**Figure 1:**
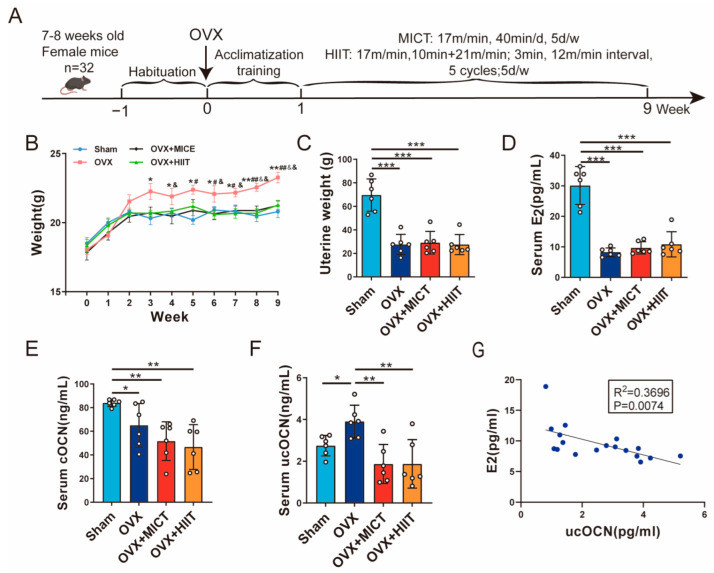
Effects of moderate-intensity and high-intensity interval treadmill on body weight, uterus weight, and serum hormones in ovariectomized mice. (**A**) Experimental timeline. (**B**) Body weight. (**C**) Uterus weight. (**D**) Serum E_2_ (**E**) Serum cOCN. (**F**) Serum ucOCN. (**G**) Correlation analysis between serum E_2_ and ucOCN (Pearson correlation analysis). *n* = 6 biologically independent samples. *** *p* < 0.001, ** *p* < 0.01, * *p* < 0.05. figure (**B**), ** *p* < 0.01, * *p* < 0.05 vs. Sham group; ^##^ *p* < 0.01, ^#^ *p* < 0.05 vs. OVX + MICT group; ^&&^ *p* < 0.01, ^&^ *p* < 0.05 vs. OVX + HIIT group. Data are represented as means ± SD.

The wet uterine weights and body weights of each group are shown in Figure 1B,C. Compared with the Sham group, the uterine weights of the OVX group, as well as the OVX + MICT and OVX + HIIT groups, were significantly reduced. There were no significant differences between the OVX group and the OVX + HIIT or OVX + MICT groups. Additionally, the body weights of the mice in the OVX group were significantly higher than those in the Sham group at the third week post-OVX, and both intensities of exercise significantly inhibited the weight gain in the OVX mice.

### 2.2. Serum E_2_, cOCN, and ucOcn Level

Serum E_2_, cOCN, and ucOCN levels were measured to assess the effects of varied exercise intensities on mice (Figure 1D–F). Compared with the Sham group, serum E_2_ levels significantly decreased in the OVX, OVX + MICT, and OVX + HIIT groups, with no significant differences among them (Figure 1D). As shown in Figure 1E,F, compared with the Sham group, the serum levels of cOCN in OVX mice were significantly reduced, while the concentrations of ucOCN were significantly increased. However, compared with the OVX group, serum ucOCN levels were significantly decreased in both the OVX + MICT group and the OVX + HIIT group. Therefore, exercise did not improve the effects of ovariectomy on serum E_2_ levels but significantly decreased the levels of serum ucOCN.

### 2.3. Lipid Parameters, Blood Pressure, and Blood Vessel Morphology

**Figure 2:**
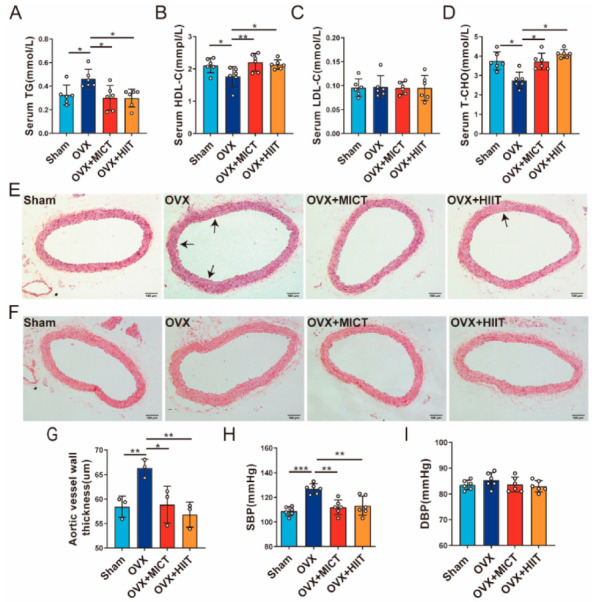
Effects of moderate-intensity continuous exercise and high-intensity interval exercise on lipid parameters, aortic morphology, aortic wall thickness, and blood pressure in mice. (**A**) Serum triglycerides. (**B**) Serum high-density lipoprotein cholesterol. (**C**) Serum low-density lipoprotein cholesterol. (**D**) Serum total cholesterol. (**E**) HE staining of the aorta, with arrows indicating rupture of elastic fibers in the blood vessels (Scale bar: 100 μm). There are multiple ruptures of elastic fibers in the aorta of OVX mice, while MICT and HIIT significantly improved the elastic fiber ruptures. (**F**) Von Kossa staining of the aorta (Scale bar: 100 μm). No significant calcification was observed in the aorta of mice in all groups. (**G**) Thickness of the aortic blood vessel wall. (**H**) Systolic blood pressure. (**I**) Diastolic blood pressure. *n* = 3/6 biologically independent samples. *** *p* < 0.001, ** *p* < 0.01, * *p* < 0.05. Data are represented as means ± SD.

As shown in Figure 2A,B, compared to Sham mice, OVX mice exhibited significantly elevated serum TG and significantly decreased HDL-C. Both types of exercise significantly reduced serum TG and increased HDL-C in OVX mice. There were no significant differences in LDL-C among the groups (Figure 2C). The changes in T-CHO were similar to HDL-C across the groups (Figure 2D). Figure 2E exhibits the aortic intima smoothness and elastic fiber arrangement of Sham, OVX, OVX + MICT, and OVX + HIIT mice. Sham mice showed smooth aortic intima and dense elastic fibers. OVX mice had aortic protrusions, disordered fibers, and ruptures. The vascular elastic fibers of OVX + MICT mice are arranged neatly and without ruptures, while OVX + HIIT mice have loose fibers with reduced ruptures. Von Kossa staining (Figure 2F) revealed no calcification in any group. Figure 2G shows OVX mice exhibited thicker aortic walls than Sham, while both exercise groups exhibited thinner walls. Figure 2H indicates higher SBP in OVX mice, and the elevation were inhibited by both exercise types, while DBP showed no significant changes across all groups (Figure 2I).

### 2.4. Microstructure of the Distal Femur

**Figure 3:**
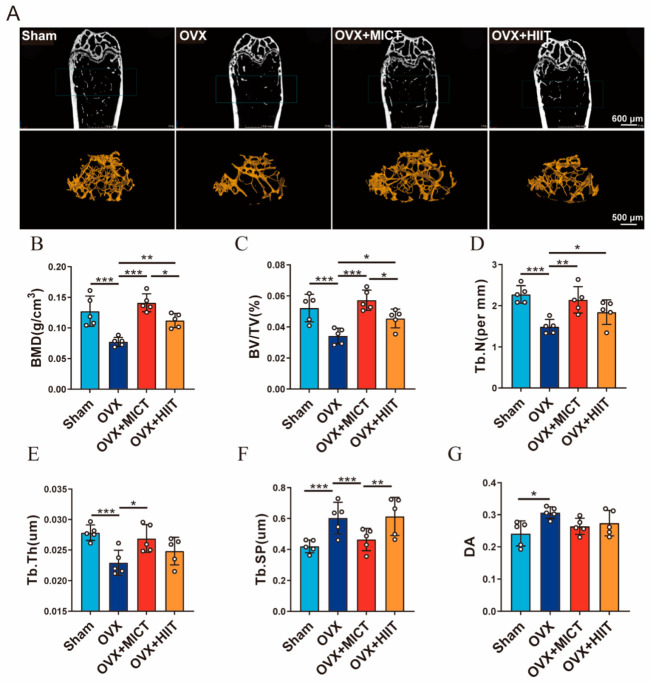
Effects of moderate-intensity continuous exercise and high-intensity interval exercise on the bone microstructure of mouse femurs. (**A**) Representative two-dimensional and three-dimensional micro-CT images of trabecular bone in the distal femur (Scale bar: 600 μm or 500 μm). (**B**–**G**) Trabecular bone microarchitecture parameters, including BMD, BV/TV, Tb.N, Tb.Th, Tb.Sp, and DA. *n* = 5 biologically independent samples. *** *p* < 0.001, ** *p* < 0.01, * *p* < 0.05. Data are represented as means ± SD.

As shown in Figure 3A, the 2D and 3D images of the distal femur revealed a significant reduction in the number of trabeculae in the cancellous bone of the distal femur region in OVX mice, accompanied by a substantial increase in trabecular spacing and deterioration of bone microarchitecture. Quantitative analysis results in Figure 3B–E show that BMD, BV/TV, Tb.Th, and Tb.N were significantly decreased in the OVX group compared to Sham mice. Compared with OVX mice, BMD, BV/TV, Tb.Th and Tb.N were significantly increased in the OVX + MICT group. Furthermore, BMD, BV/TV and Tb.N were significantly increased in the OVX + HIIT group, with no significant change in Tb.Th. Additionally, BMD and BV/TV were significantly higher in the OVX + MICT group than in the OVX + HIIT group. As shown in Figure 3F,G, compared with Sham mice, Tb.Sp and DA were significantly increased in the cancellous bone of the distal femur of OVX mice. Compared with OVX mice, Tb.Sp was significantly decreased in the OVX + MICT group, while there was no significant improvement in Tb.Sp in the OVX + HIIT group. Neither type of exercise exhibited a significant effect on DA. These results indicated that MICT on improving the microarchitecture of cancellous bone in mice was superior to HIIT, as evidenced by significantly increased BMD and BV/TV and significantly reduced Tb.Sp compared to HIIT.

### 2.5. The Number of Osteoblasts and Osteoclasts in the Tibia

**Figure 4:**
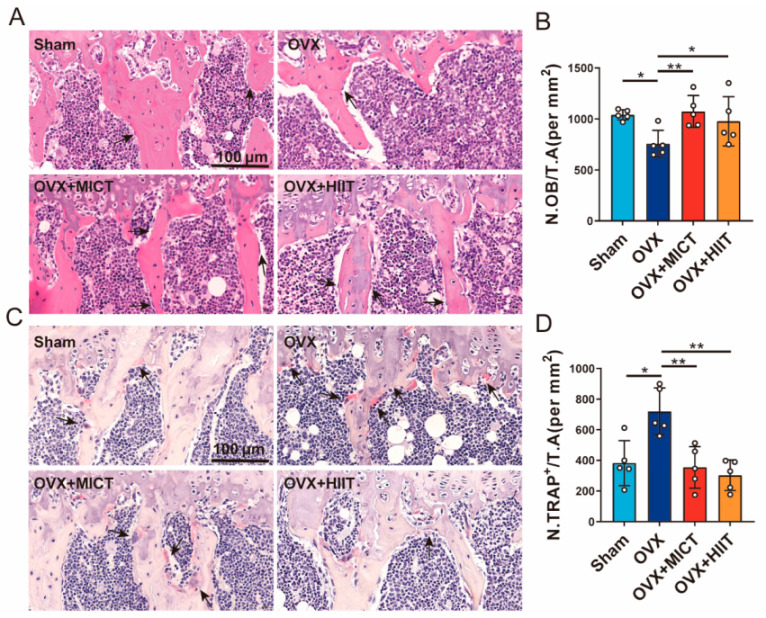
The effects of moderate-intensity continuous exercise and high-intensity interval exercise on the number of osteoblasts and osteoclasts in the proximal tibiae of mice. (**A**) HE staining of the proximal tibiae; the arrows indicate osteoblasts (Scale bar: 100 μm). (**B**) The number of osteoblasts per unit area of trabecular bone. (**C**) TRAP staining of the proximal tibiae; the arrows indicate osteoclasts (Scale bar: 100 μm). (**D**) The number of osteoclasts per unit area of trabecular bone. Ten fields of view were randomly selected for observation in each group. *n* = 5 biologically independent samples; 400× magnification. ** *p* < 0.01, * *p* < 0.05. Data are represented as means ± SD.

As shown in Figure 4A,B, the OVX group exhibited significantly fewer osteoblasts per unit area. Both MICT and HIIT exercises increased osteoblasts. As shown in Figure 4C,D, the number of osteoclasts per unit area significantly increased in OVX mice, while both exercises markedly reduced the number of osteoclasts.

## 3. Discussion

Menopause is an inevitable life stage for women, during which the aging of ovaries leads to a decrease in estrogen levels, thereby increasing the risk of various diseases [20]. Osteoporosis and cardiovascular disease are common among middle-aged and elderly women, with cardiovascular disease being the primary cause of death among older women [21,22]. Multiple studies have shown that the risk of cardiovascular disease increases after the onset of menopause [23]. Abnormal blood pressure and lipid parameters are risk factors for cardiovascular disease (CVD) [24,25]. Studies have shown that reducing systolic and diastolic blood pressure can decrease the risk of CVD [26]. Both epidemiological and experimental studies indicate that decreased HDL-C and increased LDL-C increase the risk of CVD [27,28,29]. Clinical studies demonstrated that actively improving abnormal blood lipid levels can regress atherosclerotic plaques and reduce the incidence of CVD [30]. The decline in estrogen levels in postmenopausal women and ovariectomized animals leads to elevated blood pressure and abnormal lipid parameters [31,32,33]. In our study, OVX mice exhibited significantly increased systolic blood pressure, decreased HDL-C levels, and increased TG levels. Additionally, these mice showed increased elastic fiber rupture and elevated aortic wall thickness. Both exercise intensities effectively lowered blood pressure, improved the morphological structure of the aortic wall, and reduced wall thickness. These changes might be related to the regulation of lipid parameters by exercise. Moreover, both exercise intensities significantly reduced serum TG levels and increased HDL-C levels. Notably, T-CHO levels decreased in OVX mice and increased in the exercise groups. This might be related to the lack of significant changes in LDL-C levels among the groups and the increase in HDL-C levels due to exercise. The specific mechanisms require further investigation. In summary, the four key factors significantly reducing the risk of cardiovascular diseases are attributed to exercise, including the decrease in TG levels, the increase in HDL content, the reduction in blood vessel wall thickness, and the effective control of SBP. These positive physiological changes not only effectively decrease the deposition of lipids on the inner wall of blood vessels but also enhance the cholesterol reverse transport mechanism, thereby significantly improving blood vessel elasticity and compliance, optimizing the circulatory system, and greatly reducing the burden and potential damage to the heart and blood vessels [34,35]. This series of comprehensive effects constructs a solid defense against the occurrence and development of atherosclerosis, thereby substantially lowering the incidence of cardiovascular diseases [34,35].

It is well-documented that weight gain is a common occurrence in OVX mice, primarily due to metabolic alterations resulting from decreased estrogen levels [36]. In alignment with previous studies, our results showed that the body weights of OVX mice were significantly higher than those of the Sham group [37]. This weight gain can influence cardiovascular risk factors through various mechanisms, such as increased release of inflammatory cytokines from adipose tissue and negative impacts on insulin sensitivity [38]. Interestingly, both MICT and HIIT significantly curbed the weight gain in OVX mice. This observation suggests that exercise may counteract weight gain by improving energy balance and metabolic regulation [39]. Specifically, exercise could achieve this by increasing energy expenditure, boosting basal metabolic rate, and enhancing insulin sensitivity [40].

In our previous research, exercise significantly elevated the serum osteocalcin levels in VCD-induced ovarian senescent mice and ameliorated their anxiety-like behaviors [41]. Also, studies reported that OCN is involved in the regulation of glucose and lipid metabolism and is associated with vascular atherosclerosis and vascular calcification [14]. Therefore, in this study, we measured the circulating OCN levels in mice at the 9th week after OVX and found that the levels of ucOCN were significantly elevated, while the levels of cOCN were significantly decreased. Both types of exercise significantly reduced the ucOCN levels in OVX mice but had no significant effect on cOCN levels. The significant negative relationship between OCN and estrogen indicated that changes in OCN were induced by estrogen. In fact, we observed a trend of increased estrogen levels in mice from the exercise groups. According to research reports, Wistar female rats that underwent ovariectomy exhibited a significant increase in serum estrogen levels after engaging in exercise for an extended period of time (1 h/d, 6 d/w) for a duration of three months [42]. Meanwhile, we did not detect a correlation between OCN and TG, HDL-C, SBP, or vascular wall thickness, indicating that the reduction in cardiovascular risk factors by both exercises in OVX mice is not directly related to changes in serum ucOC levels. Similar to our results, Wieczorek-Baranowska and his colleagues reported that 8 weeks of aerobic training in postmenopausal women significantly improved central obesity, decreased OCN levels, and reduced insulin resistance, but they did not observe a direct relationship between OCN concentration changes with training and metabolic markers [43]. Another study has demonstrated that there are significant gender differences in the impact of exercise on the regulation of OCN [44]. In this study, exercise increased the level of circulating OCN in female mice but decreased it in male mice. Notably, this change was associated with improvements in cognitive outcomes yet had no correlation with metabolic outcomes. Meanwhile, although exogenous osteocalcin did not improve metabolism, it had a significant effect on improving cognitive defects induced by a high-fat diet. Furthermore, some studies have reported that OCN-knockout mice did not exhibit significant insulin resistance or glucose and lipid metabolism disorders [45]. In contrast, another study involving 39 young obese male participants randomly divided them into a control group and an exercise group, with the exercise group undergoing an 8-week aerobic exercise training program [46]. The results showed that exercise-induced reduction in body fat and improvement in insulin sensitivity were accompanied by a significant increase in serum osteocalcin levels. Moreover, the increase in osteocalcin was negatively correlated with changes in body weight, BMI, body fat percentage, and insulin resistance index [46]. Therefore, it can be concluded that the impact of exercise on osteocalcin and its role in metabolic regulation may vary due to age and gender differences. Additionally, different animal models and exercise interventions of varying intensities can produce different results. Thus, more systematic and comprehensive studies, including gain-of-function and knockout experiments, are needed in the future to further verify the role of osteocalcin in the regulation of energy metabolism and cardiovascular risk factors by exercise. The regulatory effect of exercise on estrogen levels may be an important mechanism by which it improves cardiovascular risk factors in OVX mice. Meanwhile, ucOCN does not seem to be the direct cause of the improvement in cardiovascular risk factors due to exercise; rather, it may serve as a potential biomarker for assessing the effectiveness of exercise in the prevention and treatment of osteoporosis.

The microstructure of bone tissue can effectively reflect the health status of bones, and BV/TV, Tb.Th, Tb.Sp, and Tb.N are the primary indicators reflecting bone microstructure [47]. In this study, significant decreases were observed for BMD, BV/TV, Tb.N, and Tb.Th in the distal cancellous bone region of the femur in OVX mice. Additionally, Tb.Sp and DA have increased significantly. MICT was found to improve the bone microstructure more effectively than HIIT because MICT well improved BMD, BV/TV, and Tb.Sp in the distal cancellous bone of the femur in OVX mice. Our results are consistent with previous research [48]. Furthermore, low-to-moderate-intensity treadmill exercise at a speed of 10 m/min can partially reverse the trabecular bone loss induced by ovariectomy. Although high-intensity treadmill exercise at 18 m/min also exhibits certain positive effects, running at the lower intensity is more effective in reducing bone loss [49]. It further supports our results that moderate-intensity exercise is more beneficial for improving bone health. This may be related to the fact that MICT is more effective in promoting osteoblast number (+10.22%) compared to HIIT. Additionally, MICT, with its moderate-intensity continuous exercise, may apply a more sustained and appropriate load stimulus to the bones. In contrast, the appropriate stimulus time that HIIT exerts on the bones may be shorter. This is also one of the reasons why MICT is superior to HIIT in improving bone microstructure. However, the specific mechanism of action still needs further in-depth research.

Morphological analysis of tibial trabecular structure reveals that both MICT and HIIT can significantly increase the number of osteoblasts (+42.07% and +29.41%) and decrease the number of osteoclasts (−50.83% and −57.91%). Osteoblasts are key cells responsible for bone formation and bone matrix synthesis. In this study, MICT and HIIT effectively promoted osteoblast proliferation, accelerating bone formation and repair processes. Notably, MICT exhibited particularly prominent effects in this regard, which partly explains why MICT outperforms HIIT in improving bone microstructure. Osteoclasts are responsible for bone resorption and remodeling. Both MICT and HIIT significantly reduced the number of osteoclasts, indicating that these two exercise modes can inhibit the bone resorption process and reduce bone loss. Research indicates that cOCN, due to its structural characteristic of having two carboxyl groups at the termini, exhibits a strong binding ability to Ca^2+^ on the surface of hydroxyapatite, enabling it to effectively deposit in bone matrix [50]. Approximately 60% to 90% of cOCN is deposited in bone matrix, while the remaining portion is released into the circulatory system [51]. In contrast, ucOCN has a weaker affinity for bone and is more abundant in the bloodstream [52]. Notably, osteoclast activity creates acidic resorption lacunae where OCN deposited in the bone matrix may undergo decarboxylation, thereby increasing ucOCN levels in the blood [52]. Therefore, the reduction in osteoclast numbers due to exercise may contribute to lowering circulating ucOCN levels by minimizing this decarboxylation process. Despite the promotion of osteoblast proliferation by both MICT and HIIT, circulating cOCN levels do not increase, implying enhanced deposition of cOCN within the bones. Further research is needed to explore the role of OCN carboxylation in bone health and its potential implications for the prevention and treatment of bone diseases.

## 4. Materials and Methods

### 4.1. Animals

Twenty-four female C57BL/6J mice aged 7–8 weeks (purchased from the Animal Center of the Medical School of Xi’an Jiaotong University, SCXK2012-003) were housed in a sterile animal room at the School of Life Sciences and Technology, Xi’an Jiaotong University. One week after acclimatization, the experiments commenced. The mice were randomly divided into four groups: Sham group (Sham, *n* = 8), ovariectomized control group (OVX, *n* = 8), ovariectomized + moderate-intensity continuous training group (OVX + MICT, *n* = 8), and the ovariectomized + high-intensity interval training group (OVX + HIIT, *n* = 8). During the experimental period, the mice had free access to standard rodent feed and sterile water. The relative temperature and humidity in the animal room were maintained at 22 °C ± 2 °C and 60% ± 5%, respectively. The diurnal cycle was controlled at 12 h of light and 12 h of darkness. The entire procedure was reviewed and approved by the Biomedical Ethics Committee of the Medical School of Xi’an Jiaotong University in accordance with ethical principles, with an approval number of 2020-625. It was carried out in compliance with the “Guide for the Care and Use of Laboratory Animals” published by the National Institutes of Health (NIH Publication No. 8023, revised in 1978).

### 4.2. Ovariectomy

For the OVX mice, a bilateral dorsal incision surgery was performed. A vertical incision was made on each side of the midline of the back, approximately one finger’s width away from the midline, at the point between the iliac bone and the ribs. The skin and subcutaneous fascia were cut open with scissors, and the abdominal muscles were cut along the edge of the erector spinae muscles. The abdominal cavity was opened with forceps supporting the fat, which was carefully lifted out. Upon locating the ovaries, the blood vessels and fat below them, as well as the uterus, were suture-ligated, and the ovaries were removed. The muscles and skin were sutured layer by layer, and the wound was disinfected with iodophor. For the Sham group mice, their ovaries were retained, and only an equivalent volume of fat next to the ovaries was removed. The wounds of the mice recovered well after the surgery, and no infections occurred.

### 4.3. Exercise Protocols

The two exercise groups of mice underwent an adaptive training period of 6 min per day at a speed of 6 m/min for three consecutive days. Following the adaptive training, the maximum running capacity (MRC) of the mice was measured. The measurement method involved starting at an initial speed of 6 m/min, with an increase of 3 m/min every 3 min, until the mice could no longer keep up with the treadmill speed [53], with a maximum detectable speed of 24 m/min. The exercise protocol for the OVX + MIIT group was as follows: continuous exercise at 70% of the MRC (17 m/min) for 40 min/d [53], five days per week, for a duration of 8 weeks. The exercise protocol for the OVX + HIIT group was as follows: intermittent exercise consisting of 90% and 50% of the MRC [53]. Specifically, each day began with 10 min of exercise at 17 m/min, followed by five cycles of 3 min at 21 m/min interspersed with 3 min at 12 m/min, five days per week, for a duration of 8 weeks. All exercise sessions were completed between 6:00 p.m. and 9:00 p.m.

### 4.4. Blood Pressure Measurement

Blood pressure in mouse tails was measured using a small animal blood pressure monitor (BP-2010A, Reward, Beijing, China). The mouse was restrained using a fixing device to expose its tail, which was then placed in a heated chamber at 37 °C to allow the mouse to acclimate to the environment. The pressure cuff of the blood pressure measuring device was placed near the base of the mouse’s tail. Blood pressure measurement began once the mouse had calmed down. Each animal was measured 20 times, and the average of the middle ten readings was taken as the mouse’s blood pressure value.

### 4.5. Serum Analysis

Strictly adhering to the instructions provided by the commercial ELISA kits, we assessed the levels of estradiol (E_2_, Cloud-Clone Corp, Wuhan, China), Gla-osteocalcin (cOCN, Takara, Tokyo, Japan), Glu-osteocalcin (ucOCN, Takara, Tokyo, Japan), triglyceride (TG, Nanjing Jiancheng, Nanjing, China), high-density lipoprotein cholesterol (HDL-C, Nanjing Jiancheng, Nanjing, China), low-density lipoprotein cholesterol (LDL-C, Nanjing Jiancheng, Nanjing, China), and total cholesterol (T-CHO, Nanjing Jiancheng, Nanjing, China). A Bio-Rad Model 680 microplate reader (Bio-Rad, Hercules, CA, USA) was utilized to measure the absorbance values. The detection thresholds were established at 12.35 pg/mL for E_2_, 10.5 ng/mL for cOCN, and 0.25 ng/mL for ucOCN.

### 4.6. Morphometric Analysis

The aorta and tibia were dissected and fixed with 4% formaldehyde. The tibia was then decalcified, sectioned, and prepared for embedding in wax after washing with PBS. The aorta underwent dehydration and wax embedding. Both were sectioned at 8–10 μm, mounted on slides, and stained with hematoxylin and eosin. For Von Kossa staining, sections were washed, immersed in silver solution, exposed to light, treated with sodium thiosulfate, counterstained with Van Gieson’s stain, and then processed for dehydration, clearing, and mounting. Histophysiological evaluations were conducted under a microscope. Aortic structure and thickness were measured at 200× magnification, while osteoblast and osteoclast count per trabecular unit area were calculated at 400× magnification.

### 4.7. Micro-CT Analysis

**Figure 5:**
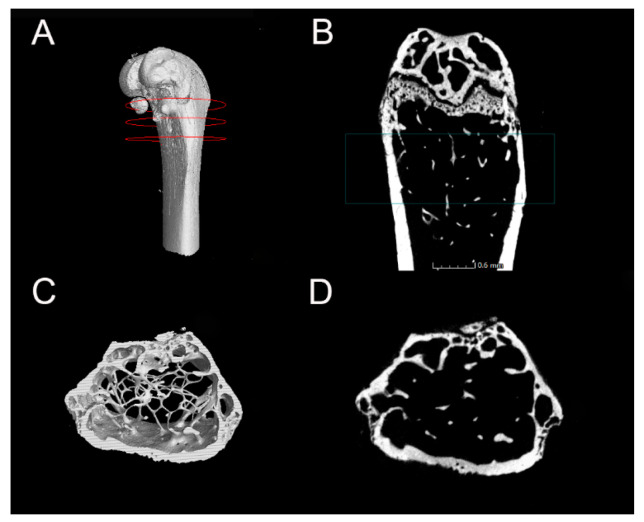
The region of interest (ROI) in the distal femur of mice. (**A**) 3D image of the ROI area. (**B**) Two-dimensional image of the ROI area. (**C**) Top-down 3D view of the ROI. (**D**) Top-down 2D view of the ROI.

The microarchitecture of the trabecular bone region in the distal femur of mice was scanned using the German Y. Cheetah micrometer X-ray three-dimensional imaging system (Y.Cheetah; YXLON International GmbH, Hamburg, Germany). The parameters were set as follows: voltage at 80 KV, current at 35 μA, resolution at 6 μm, and a total of 720 scanning layers. After the scanning was completed, the grayscale images obtained from X-ray imaging were reconstructed. Using VG Studio MAX 3.0 analysis software, the region of interest (ROI) was selected. Based on the anatomical features of the femur, the first layer where both the medial and lateral condyles of the distal femur simultaneously disappeared was identified, and a cylinder with a radius of 1.5 cm and a height of 1 cm was selected as the ROI, extending from bottom to top. Trabecular bone within this region was extracted for analysis. This process yielded visual 2D and 3D images (Figure 5) and quantitative indices of the trabecular microarchitecture of the distal femur, including bone mineral density (BMD), bone volume fraction (BV/TV), trabecular thickness (Tb.Th), trabecular number (Tb.N), trabecular separation (Tb.Sp), and degree of anisotropy (DA).

### 4.8. Statistical Analysis

The results are presented as mean ± standard deviation (mean ± SD). Statistical analyses were conducted using SPSS version 20.0 software (SPSS Institute, Chicago, IL, USA). All data were tested using the one-sample Kolmogorov–Smirnov test and were found to be normally distributed. One-way analysis of variance (ANOVA) was employed to assess whether there were differences among the three groups. Once a significant difference was detected, the least significant difference multiple comparison test was used to determine whether the difference between every two groups was statistically significant. *p*-value < 0.05 was considered statistically significant.

## 5. Conclusions

In summary, both MICT and HIIT can effectively improve the cardiovascular disease-related risk factors in OVX mice, but moderate-intensity treadmill exercise much more enhances bone mineral density and improves bone microstructure in OVX mice. It means that in postmenopausal women, opting for MICT appears to be more beneficial to both the cardiovascular system and the skeletal system. UcOCN could serve as a metabolic biomarker for improvements of bone health through exercise, but it has not been found to participate in the regulation of cardiovascular disease-related risk factors, at least in the current study.

## Data Availability

The data underlying this article will be shared upon reasonable request to the corresponding author.
